# Preoperative anemia and perioperative blood transfusion in head and neck squamous cell carcinoma

**DOI:** 10.1371/journal.pone.0205712

**Published:** 2018-10-22

**Authors:** Philipp Baumeister, Martin Canis, Maximilian Reiter

**Affiliations:** 1 Department of Otorhinolaryngology, Head and Neck Surgery, Ludwig-Maximilians-University, Munich, Germany; 2 Clinical Cooperation Group *Personalized Radiotherapy in Head and Neck Cancer*, Helmholtz Center, Munich, Germany; University of Saskatchewan College of Medicine, CANADA

## Abstract

**Objectives:**

To evaluate the impact of preoperative anemia and perioperative blood transfusion (PBT) on disease free (DFS) and overall survival (OS) of patients with head and neck squamous cell carcinoma (HNSCC).

**Methods:**

Retrospective study of 354 patients primarily treated with surgery between 2006 and 2016. Cases were selected according to completeness and accuracy of available clinical data. Thus, a selection bias cannot be excluded. Patients who received PBT were identified by our controlling department and verified by our blood bank data base.

**Results:**

Both, preoperative anemia and PBT significantly decreased OS in univariate analysis. Although PBT was needed more frequently by older patients in worse physical conditions with more advanced HNSCC, subgroup analysis also demonstrate a profoundly negative effect of PBT on OS in younger patients and early stage HNSCC. According to a restrictive transfusion policy at our hospital the transfusion rate was comparably low. We could not verify increasing effects of PBT on cancer recurrence rates as it was previously shown.

**Discussion:**

Preoperative anemia is the most common paraneoplastic syndrome in HNSCC. Despite its devastating prognostic effect we suggest a restrictive transfusion policy whenever possible. Our data also show that anemia as an independent prognostic factor in head and neck surgical oncology is defined not only by low hemoglobin concentrations but low red blood cell counts as well.

## Introduction

There is an increasing awareness of the negative effects of blood products in cancer treatment [1]. Once considered as a benign intervention, perioperative blood transfusion (PBT) is nowadays recognized as a measure that should be avoided if possible. Seemingly purified products like red blood cell (RBC) suspensions contain variable contents of other blood cells and biologically active compounds [2, 3]. Several processes that occur during and after transfusion are discussed as causes for its adverse effects in surgical oncology. As reviewed by Cata *et al*., these mechanisms include immunosuppression induced by and inflammatory response to PBT. Before routine depletion of leucocytes, post-transfusion infections and febrile reactions were common adverse events [4]. By leucoreduction, the release of bioactive substances from white blood cells can largely be avoided. Nevertheless, several actives cytokines are still present in stored erythrocyte units [5]. Units of red blood cells also contain lipids and a variety of pro-inflammatory mediators like phosphatidylcholines and eicosanoids, as well as microparticles containing numerous growth factors [6, 7, 8]. The immunomodulatory effects of allogeneic blood transfusions include a decrease of interleukin 2 secretion, a diminished activity of natural killer cells and macrophages, as well as hypersensitivity reaction. PBT can undoubtedly save lifes. On the other hand, mounting evidence shows that it negatively affects patient`s immune surveillance of cancer and promotes tumor growth and dissemination [4, 8].

Numerous systematic reviews and meta-analyses prove the adverse influence of PBT on outcomes in literally all solid cancers including cholangiocarcinoma, liver, bladder, pancreatic, gastric and lung cancer [9–14]. However, much less is known about PBT`s role in head and neck squamous cell carcinoma (HNSCC). In contrast to, for example, hepatectomy, intra- and perioperative blood transfusions are relatively rare events in the surgical treatment of HNSCC. Still, preoperative anemia is common among HNSCC patients. Anemic conditions before treatment may be attributed to the disease itself, impaired dietary intake and comorbid conditions of HNSCC patients [15–17].

We recently described the negative impact of anemia on prognosis of oropharyngeal cancer patients [18]. In this localization two distinct HNSCC entities need to be distinguished. On one hand there are “classic” carcinomas linked to tobacco and alcohol consumption and with a peak onset within the 6^th^ decade of life. Affected patients often show a variety of comorbid diseases associated with the respective noxious agents and age. On the other hand an increasing number of oropharyngeal carcinomas are caused by a persistent infection with high-risk human papilloma viruses (HPV). Affected patients are often younger, have no or a limited smoking history and, therefore, less comorbid diseases. Clinically, HPV-associated HNSCC in the oropharynx are identified by positive immunohistochemical staining of p16, a cell cycle regulator that accumulates within cancer cells in the course of HPV-driven carcinogenesis. Despite being less differentiated and showing more advanced cervical lymph node metastasis, HPV-/p16-positive HNSCC are characterized by a remarkably improved therapeutic response and patient`s survival. Less exposure to tobacco-contained carcinogens, less genetic mutations and better overall health are discussed as major contributors to favorable prognosis. Distinct etiology, tumor biology and therapeutic response led to the acknowledgement of p16-positive oropharyngeal squamous cell carcinomas as a discrete cancer entity in the head and neck with a separate clinical staging system valid since January 2017 [19, 20]

For this study we extended the cohort and included patients who received PBT during their stay at our department. The aim of the study was to confirm our previous results regarding the prognostic effects of anemia in HNSCC and to evaluate the impact of PBT on survival in these patients.

## Methods

We retrospectively included 354 patients who underwent surgical resection of their HNSCC at the Department of Otorhinolaryngology, Head and Neck Surgery, Ludwig-Maximilians-University (LMU), Munich, between January 2006 and October 2016. For this period we obtained a list of patients from our controlling department who were administered blood products. This list was then matched with our blood bank to identify patients who received PBT. All clinical data was obtained from paper based and digital patient files. Follow up data was recorded during posttherapeutic visits and from The Munich Cancer Registry. Clinical data was initially collected without involving the ethics committee, LMU, Munich. After subsequent consultation, the committee stated that patients are not required to sign an informed consent and endorses the publication of the manuscript. A minimal, fully anonymized set of clinical data is available as supporting information.

The *World Health Organization* (WHO) defines anemia as “*a condition in which the number of red blood cells or their oxygen-carrying capacity is insufficient to meet physiologic needs*, *which vary by age*, *sex*, *altitude*, *smoking*, *and pregnancy status”* (http://www.who.int/topics/anaemia/en/). The *WHO Global Database on Anemia* reports on the worldwide prevalence of anemia based on hemoglobin concentration. We used both, red blood cell counts (RBC) and hemoglobin-concentration to define anemic conditions as:

1. RBC <4.3T/l in women and <4.8T/l in men, **and/or**
2. hemoglobin-concentration <12 g/dL in women and <14 g/dL in men

Although the WHO uses a worldwide threshold of 13 g/dL for men >15 years of age below which the individual is considered to be anemic, the medical center of Ludwig-Maximilians-University Munich applies the local population based threshold of 14 g/dl for adult men [21]. The hemoglobin threshold that triggers blood transfusion in head and neck surgical oncology is 7 g/dl unless comorbid diseases require other measures. Therefore, our clinic pursues a restrictive transfusion policy [22].

Erythrocytes concentrates transfused within the observation time were obtained from the *Bavarian Red Cross*, Munich, Germany or from the *Institute for Transfusion Medicine*, Suhl, Germany. The major additive during a maximum storage period of 49 days was mannitol. All erythrocyte concentrates/units of packed red blood cells underwent leucocyte depletion before storage.

The perioperative period was defined as the timespan between inpatient admission before surgery and discharge from our department. Disease free survival was defined as the time span between surgery and disease recurrence (local and/or regional and/or distant, as well as second primary carcinomas) or death of any cause. Overall survival was defined as the period of time between cancer resection and death of any cause.

Statistical analysis was performed using IBM SPSS Statistics, version 24. p-level was set to 0.05. To test relations between nominal and ordinal scaled factor, the chi square test was applied. Metric scaled variables were tested using the Mann-Whitney-U test. Survival estimates were produced with the Kaplan-Meier method. Significant differences were calculated using log rank test in univariate and cox regression for multivariate analysis. The number of variables included for multivariate analysis were restricted to 1 per 10 events. For this purpose we selected the most significant factors in univariate analysis.

For manuscript writing we tried to adhere to *The Strengthening the Reporting of Observational Studies in Epidemiology (STROBE) Statement*: *guidelines for reporting observational studies* [23].

## Results

354 cases were included. Patient and tumor data are summarized in Table 1. All patients underwent surgical resection of their carcinoma with or without free flap reconstruction, 343 received uni- or bilateral neck dissection. 258 patients were treated with adjuvant (chemo)radiotherapy following surgery. To avoid bias regarding intraoperative blood loss, we excluded tumor resection carried out with lasermicrosurgery. Thus, only total laryngectomies were included.

**Table 1:** Clinical data.

		n (%)
***Preoperative anemia***	yes	154 (43.5%)
	no	200 (56.5%)
***Perioperative blood transfusion***	yes	65 (18.4%)
	no	289 (81.6%)
***Age***	<60	171 (48.3%)
	≥60	183 (51.7%)
***ASA (physical status)***	ASA1	19 (5.4%)
	ASA2	152 (42.9%)
	ASA3	179 (50.6%)
	ASA4	1 (0.3%)
	N/A	3 (0.8%)
***Smoking***	current	187 (52.8%)
	former	77 (21.8%)
	never	75 (21.2%)
	N/A	15 (4.2%)
***Localization***	oral cavity	93 (26.3%)
	oropharynx	221 (62.4%)
	hypopharynx	34 (9.6%)
	larynx	6 (1.7%)
***pT-stage***^1^	pT1	95 (26.8%)
	pT2	145 (41.0%9
	pT3	90 (25.4%)
	pT4a	24 (6.8%)
***pN-stage***^1^	pN0	126 (35.6%)
	pN1	55 (15.5%)
	pN2a	28 (7.9%)
	pN2b	80 (22.6%)
	pN2c	49 (13.8%)
	pN3	5 (1.4%)
	pNx	11 (3.1%)
***UICC-stage***^1^	I	47 (13.3%)
	II	53 (15.0%)
	III	97 (27.4%)
	IVA	141 (39.8%)
	IVB	4 (1.4%)
	N/A	11 (3.1%)
***p16***	positive	71 (20.1%)
	negative	87 (24.6%)
	N/A	201 (55.4%)
***Perineural invasion***	Pn0 (absent)	106 (29.9%)
	Pn1 (present)	33 (9.3%)
	N/A	139 (39.3%)
***Lymphovascular invasion***	L0 (absent)	113 (31.9%)
	L1 (present)	68 (19.2%)
	N/A	173 (48.9%)
***Vein invasion***	V0 (absent)	166 (46.9%)
	V1 (present)	12 (3.4%)
	N/A	176 (59.7%)
***Extranodal extension***	ENE-	63 (17.8%)
	ENE+	58 (16.4%)
	N0^2^	126 (35.6%)
	N/A	107 (30.2%)
***Grading***	G1	24 (6.8%)
	G2	134 (37.9%)
	G3	196 (55.4%)
***Adjuvant treatment***	yes	258 (72.9%)
	no	95 (26.8%)
	N/A	1 (0.3%)

^1^ Union International contre le Cancre (UICC), Staging Manual, 7^th^ edition;

^2^ clinically or pathologically N0

In accordance to what is currently known about p16-negative and -positive HNSCC, all 71 p16-positive tumors were located in the oropharynx. Patients with p16-positive tumors were less often current smokers (30.9% vs. 65.9%; p<0.001). p16-positive cancers were less differentiated (G3: 83.1% vs. 54.0%; p<0.001) and had more often spread to cervical lymph nodes (pN+: 84.5% vs. 61.4%; p = 0.001). No difference between p16-positive and -negative cases was found regarding age and preoperative assessment of physical status according to the *American Society of Anesthesiologists* (ASA).

### Anemia

Preoperative blood samples were taken at least 10 days before surgery. Mean RBC was 4.50 T/l (median: 4.53; range: 2.32–5.81), mean hemoglobin concentration 13.99 g/dl (median: 14.2; range: 8.0–17.8). A total of 154 patients (43.5%) were found to be anemic. 70 patients had low hemoglobin level and low RBC counts. 9 patients had low hemoglobin concentrations, but normal RBC counts. 75 patients were classified as anemic because of low RBC counts only.

Anemic conditions were independent from gender, age, ASA-classification, pathological tumor (pT-), pathological lymph node (pN-) stage and prognostic stage group as defined by the *Union Internationale Contre le Cancre* (UICC). Current (49.7%) or former smokers (41.6%) at the time of diagnosis were more often anemic compared to never smokers (25.3%; p = 0.001). Hence, patients diagnosed with HPV-associated, p16-positive HNSCC suffered less often from anemic conditions than p16-negative cases (26.8% vs. 60.9%; p<0.001).

### Perioperative blood transfusion (PBT)

65 patients (18.4%) received at least one PBT. In 24 of these cases (36.9%) a postoperatively developing or increasing anemia without any apparent cause was corrected. 23 patients (35.4%) received PBT during initial tumor resection or during revision surgery. In 12 cases (18.5%) at least one erythrocyte concentrate was transfused in response to postoperative bleeding. 6 patients (9.2%) needed blood transfusion(s) in the course of measures unrelated to the tumor resection. For instance, two patients suffered a postoperative myocardial infarction and needed PBT to support coronary perfusion. One patient received a total of 45 erythrocytes transfusions in the course of a fulminant deep vein thrombosis that finally led to leg amputation. A total of 11 patients received platelet concentrates only (1 patient), fresh frozen plasma only (5 patients), or a combination of both (5 patients) in addition to erythrocyte concentrates. 56.9% of transfused patients received 1–2 erythrocyte concentrates, the median amount was 2.0 units per patient (range: 1–45; average: 4.9).

The need for PBT was independent from gender. Patients who received PBT were older (62.6 vs. 59.7 years; p = 0.021) and in worse physical condition. Whereas only 4.1% of patients classified as ASA1/2 were given PBT, this rate was 31.7% in ASA3/4-patients (p<0.001). No correlation was found with smoking habits. PBT was given less often to preoperatively non-anemic patients (10.5% vs. 28.6%; p<0.001) and more often to patients with p16-negative HNSCC (33.3% vs. 16.9%; p = 0.019). Furthermore, patients with advanced pT- and pN-stages received more frequently PBT (pT1-2 vs. pT3-4a: 12.9% vs. 29.8%; p<0.001; pN0-pN2b vs. pN2c-pN3: 14.5% vs. 25.9%; p = 0.038). Transfusion rate was, thus, 7.0% for UICC-stage I /II- and 20.2% in UICC-stage III-IVb-patients (p = 0.001).

### Survival analysis

All factors shown in Table 1 were submitted to uni- and multivariate analysis of disease free (DFS) and overall survival (OS; Table 2). We recorded 128 recurrences, 126 patients died. Thus, factors included in multivariate analysis of DFS and OS were restricted to 13 (1 variable per 10 events).

**Table 2:** Survival analysis (entire cohort).

**Disease free survival**			
		univariate (log rank test)	multivariate (cox regression)
	n/censored	p	p
Preoperative anemia	354/226	**0.043**	0.792
Perioperative blood transfusion	354/226	0.137	0.496
Age	354/226	0.182	0.427
ASA	351/225	0.297	0.619
Smoking	339/217	0.628	-
Localization	354/226	**0.002**	0.055
pT-stage	354/226	0.059	**0.029**
pN-stage	343/223	**0.033**	0.566
UICC	343/223	0.825	-
p16	158/118	**0.016**	0.525
Perineural invasion	139/105	**0.002**	0.124
Lymphovascular invasion	181/134	0.438	0.365
Vein invasion	178/131	0.358	0.561
Extranodal extension	121/89	0.077	0.588
Grading	354/226	0.816	-
Adjuvant treatment	353/225	**0.032**	0.957
**Overall survival**			
		univariate (log rank test)	multivariate (cox regression)
	n/censored	p	p
Preoperative anemia	354/226	**<0.001**	0.663
Perioperative blood transfusion	354/226	**<0.001**	0.363
Age	354/226	**0.001**	0.296
ASA	351/225	**0.027**	0.778
Smoking	339/217	0.155	0.422
Localization	354/226	**0.007**	**0.014**
pT-stage	354/226	0.102	0.952
pN-stage	343/223	**<0.001**	0.598
UICC	343/223	**0.041**	0.449
p16	158/118	**0.006**	0.404
Perineural invasion	139/105	**<0.001**	**0.009**
Lymphovascular invasion	181/134	0.112	-
Vein invasion	178/131	**0.019**	0.859
Extranodal extension	121/89	0.078	0.330
Grading	354/226	0.985	-
Adjuvant treatment	353/225	0.240	-

Even though preoperative anemia influenced DFS, there were more significant factors in univariate analysis. In multivariate analysis only pT-stage remained significant. Both, preoperative anemia and PBT were highly significant for OS in univariate analysis. Also, pN-stage and perineural invasion showed comparable significance. Whereas pretreatment anemia and PBT lost significance in multivariate analysis, perineural invasion emerged as the most significant factor for OS.

**Fig 1:**
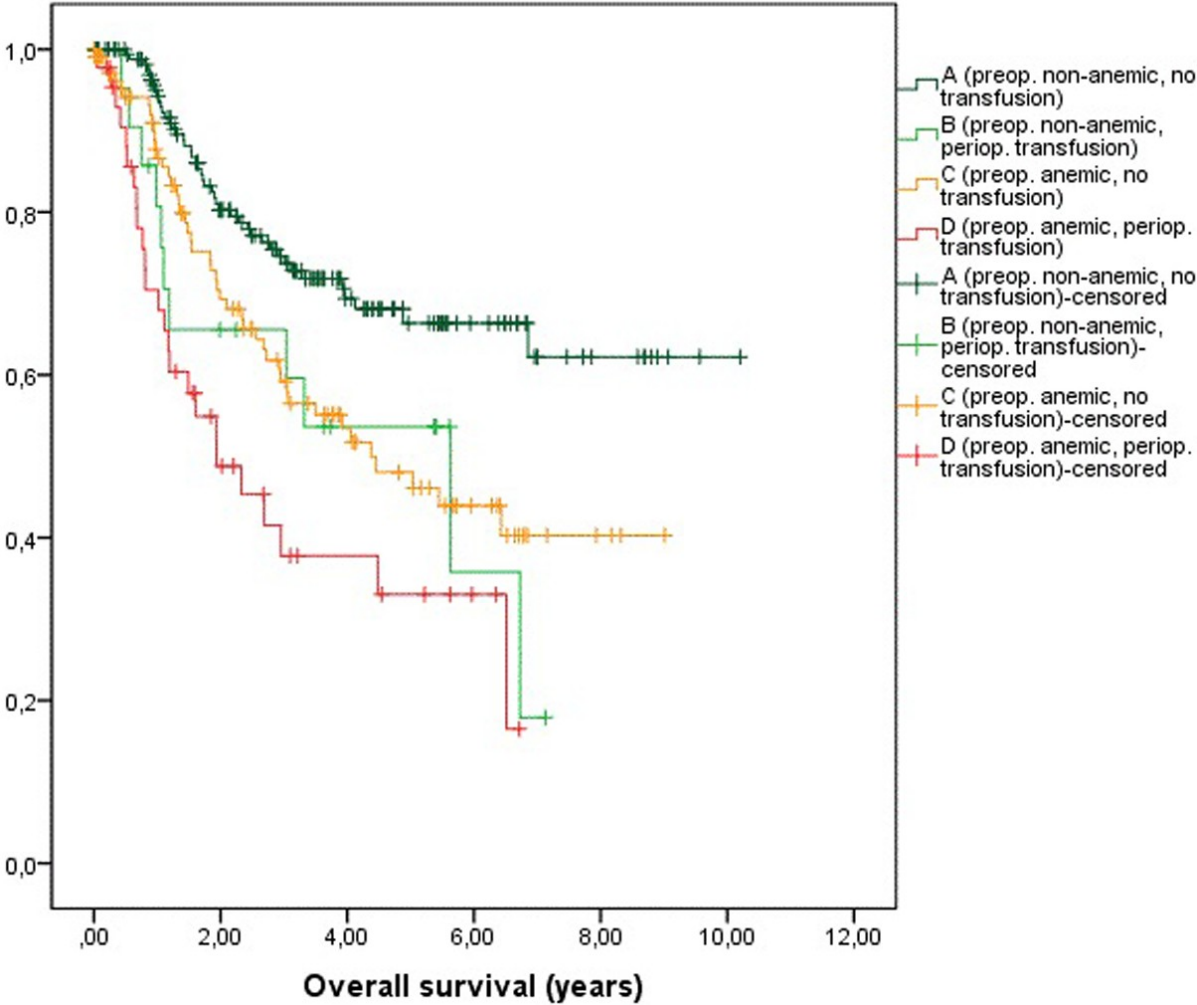
Kaplan-Meier-plots of overall survival of: A: preoperatively non-anemic, not transfused patients. B: preoperatively non-anemic, transfused patients. C: preoperatively anemic, not transfused patients. D: preoperatively anemic, transfused patients.

To emphasize the effects of preoperative anemia and PBT we build four classes (Fig 1):

- Preoperatively non-anemic patients, no transfusion (n = 179)
- Preoperative non-anemic patients, perioperative transfusion (n = 21)
- Preoperative anemic patients, no transfusion (n = 110)
- Preoperative anemic patients, perioperative transfusion (n = 44)

Preoperatively non-anemic patients had a significantly better OS compared to non-anemic patients who received PBT (A vs B, p = 0.0179), to anemic patients without PBT (A vs C, p = 0.002) and to anemic patients who needed PBT (A vs D, p<0.001). No statistical difference in OS was found between transfused non-anemic patients and anemic patients without PBT (B vs C, p = 0.687) and transfused anemic patients (B vs. D, p = 0,189), respectively. Moreover, anemic patients who received PBT had a significantly worse OS compared to anemic patients who were not transfused (C vs. D, p = 0.021).

As stated before, PBT was more frequently administered to older patients, patients in worse physical conditions, anemic patients, patients with p16-negative HNSCC and patients with more advanced pT- and pN-stage HNSCC. Because all these factors are major determinants of OS, we did an analysis of the influence of PBT on OS in 6 subgroups: In patients <60 years of age, in patients classified as ASA1 or 2, in non-anemic patients, in patients with p16-positive HNSCC and in patients with pT1-2 and pN0-2b HNSCC, respectively. Aside from ASA1 & 2-patients PBT led to a significantly worse survival in all these subgroups (Table 3).

**Table 3:** Overall survival (OS) in subgroups.

Subgroup	PBT	OS (95% CI) [years]	p (log rank test)
Age < 60	no	7.03 (6.25–7.81)	**<0.001**
	yes	2.98 (1.71–4.24)	
ASA1 & 2	no	6.93 (6.22–7.64)	0.365
	yes	5.01 (2.55–7.48)	
Normemic patients	no	7.35)6.64–8.07)	**0.017**
	yes	4.16 (2.95–5.38)	
p16-positive HNSCC	no	6.04 (5.47–6.62)	**0.042**
	yes	3.80 (2.71–4.89)	
pT1 & 2	no	6.93 (6.27–7.59)	**<0.001**
	yes	3.31 (2.36–4.26)	
pN0-2a	no	6.97 (6.36–7.59)	**0.029**
	yes	4.23 (3.29–5.17)	

**Fig 2:**
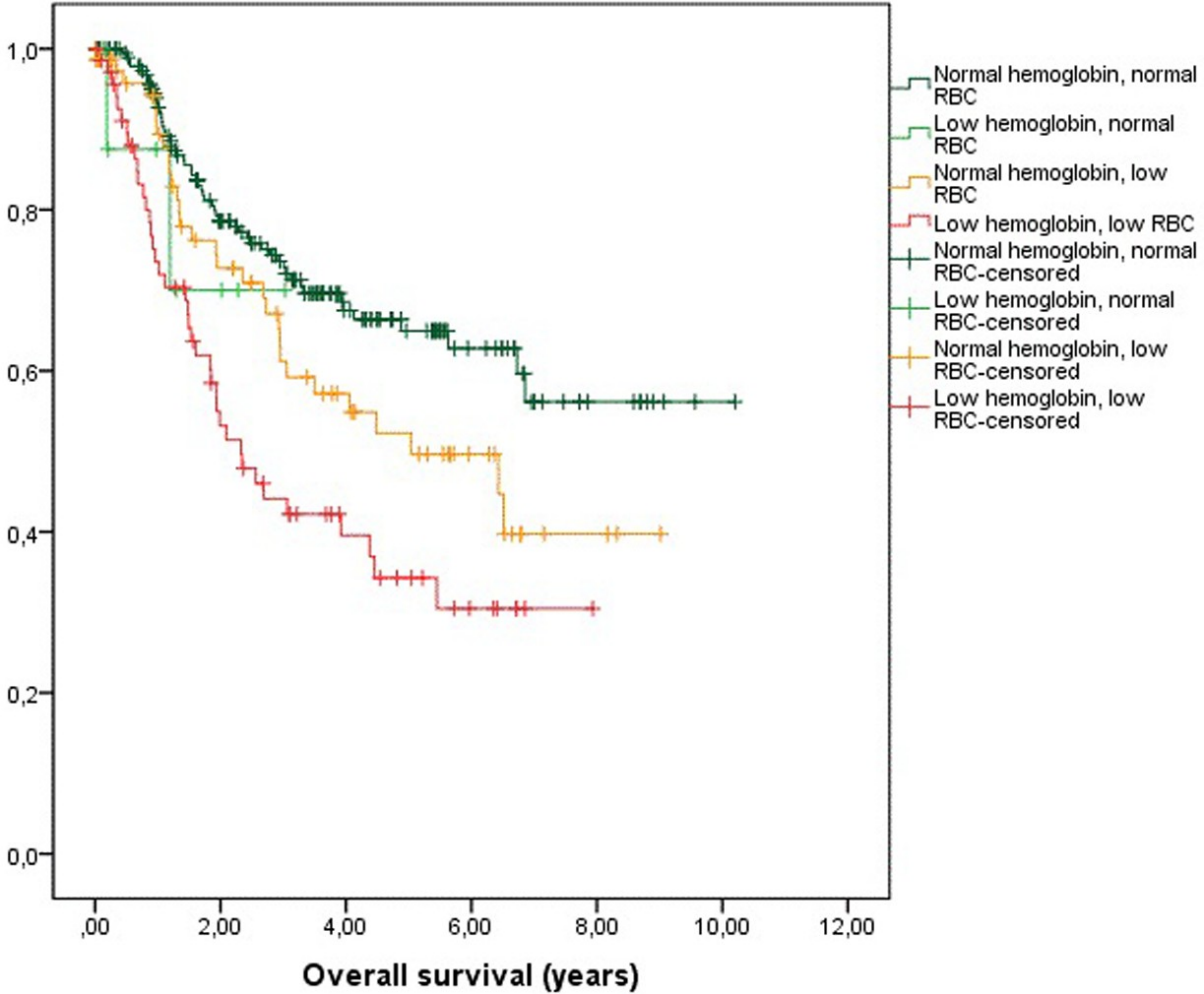
Kaplan-Meier-plots of overall survival depending on hemoglobin-levels and red blood cell (RBC)-counts.

OS depending on hemoglobin and RBC status is shown in a separated figure (Fig 2).

## Discussion

Approximately 40% of all cancer patients are anemic at diagnosis. Hence, anemia is the most common paraneoplastic syndrome. The causes of anemia in cancer include tumor-triggered formation of antibodies leading to autoimmune hemolysis. Antibodies may also suppress erythropoietin-induced RBC production causing pure red-cell aplasia. In some advanced malignancies physically injured erythrocytes, as a consequence of vascular damage and activation of coagulation by the tumor, die prematurely, causing microangiopathic hemolytic anemia. The commonest type of cancer-associated anemia and anemic conditions caused by other chronic diseases is defined by impaired iron metabolism. As a consequence of the increased expression of hepcidin, a type II acute-phase reactant, intestinal iron absorption and iron release from macrophages is downregulated. This leads to a functional iron deficiency that worsens low nutritional iron intake often seen in HNSCC patients [24–26]. Tobacco consumption, still the leading cause of carcinogenesis in the head and neck and comorbid diseases in HNSCC patients, may initially counterbalance paraneoplastic anemia due to the effects of chronic carbon monoxide exposure, but cigarette smoke is also linked to hemolysis, aplastic anemias and hematotoxicity itself [16]. As reported previously, the impact of smoking on anemia in HNSCC patients, can be demonstrated comparing patients diagnosed with p16-negative to patients suffering from p16-positive oropharyngeal carcinomas. Significantly less tobacco consumption in the latter group leads to significantly less comorbidities and, thus, to significantly less anemic conditions [18]. Accordingly, these patients are less likely to receive PBT.

Anemia is an independent prognostic factor in various solid malignancies. It was found to have an exceptionally pervasive effect in head and neck carcinomas and to lead to a relative risk of death increased by 75% compared to non-anemic cancer patients [27]. It was recently shown that preoperatively low hemoglobin levels decrease OS in a study of 167 head and neck cancer patients undergoing tumor resection followed by free flap reconstruction between July 2007 and February 2013 [28]. As demonstrated by Chau *et al*., preoperative hemoglobin concentrations <12 g/dL increased recurrence rates and survival in a retrospective study of 520 patients who underwent primary resection of various head and neck malignancies [29].

The existence of far-reaching effects of blood transfusion on the immune system is no longer subject of discussion. The condition termed transfusion-related immune modulation is well established in transfusion medicine and its immunosuppressive component was once therapeutically exploited to reduce allograft rejection after kidney transplantation [4, 30]. This very effect is suspected to increase the risk of disease recurrence and decreased survival in surgical oncology. Accordingly, PBT was shown to be associated with higher recurrence rates and decreased survival in head and neck cancer. Taniguchi *et al*. retrospectively analyzed 105 patients with stage III and IVa oral cavity squamous cell carcinomas who underwent tumor resection and neck dissection between January 1990 and December 1998. Overall survival of patients who received ≥3 units was significantly lower [31]. This finding was later confirmed by Szakmany *et al*. in a study of 559 patients with oral cavity and oropharyngeal squamous cell carcinomas undergoing primary surgery between 1992 and 2002 [32]. Danan and colleagues also supported the threshold of ≥3 units of packed red blood cells transfusion. Patients who received this amount had a significantly increased risk of death [28]. In the study by Chau and colleagues, PBT was associated not only decreased survival but also with higher recurrence rates [29]. The results of the latter studies were opposed by Fenner and colleagues who retrospectively analyzed the cases of 223 patients with oral squamous cell carcinoma undergoing primary surgery between July 1998 and June 2002. According to their results, transfusion of >4 units of packed red blood cells did not influence overall survival [33].

Interestingly, transfusion rates vastly differed between these studies. While Taniguchi and colleagues report that 61.0% of their patients received one or more PBT [31], this rate was 77.1% [32], 82.0% [28], 37.9% [29] and 97.3% [33] in other studies cited.

In our study both, peroperative anemia and PBT had little impact on DFS. In contrast, both factors significantly decreased OS in univariate analysis, even though tumor localization and perineural invasion of cancer cells emerged as most significant in multivariate analysis (Table 2). These results lead to the question whether the administration of PBT increased mortality, or whether patients who received PBT were more likely to decease anyway. Because in our study PBT was more frequently needed by older patients, patients in worse physical conditions, anemic patients, patients with p16-negative and more advanced pT- and pN-stage HNSCC, we suggest that these factors contributed to the significance of PBT in univariate analysis of OS. Also, patients who received PBT died significantly more often of cancer-unrelated causes (38.9% vs. 28.1%; p = 0.031). Nevertheless, subgroup analyses revealed the profound negative impact of PBT on OS in patients <60 years of age, non-anemic patients, patients with p16-postive HNSCC and early pT- and pN-stage carcinomas, too (Table 3). Notably, subgroup analyses cannot be interpreted as a substitute of multivariate analyses.

There is an ongoing discussion regarding adequate triggers for red blood cells transfusion. Two major transfusion strategies can be discerned. According to the liberal transfusion policy, the threshold hemoglobin concentration is 9–10 g/dL. The restrictive transfusion strategy`s threshold is 7–8 g/dL [22]. In a recent review for the Cochrane collaboration, Carson and colleagues conclude that there is good evidence that transfusions with allogeneic red blood cells can be avoided in most hospitalized patients with hemoglobin concentrations above 7 g/dL to 8 g/dL [34]. The policy of both, the Department of Anesthesiology and the Department of Otorhinolaryngology, Head and Neck Surgery in our clinic is to avoid PBT for head and neck cancer patients until a threshold of <7 g/dL is reached. Thus, different transfusion strategies are most likely the reason for vastly differing transfusion rates. Our cohort had the lowest transfusion rate of all studies cited. Because of the retrospective design of this study a selection bias cannot be excluded, but the calculated transfusion rate of 18.4% corresponds with the clinical experience of the authors.

The WHO defines anemia primarily as insufficient number of red blood cells. Applying this very definition of anemia, not only low hemoglobin concentrations as a surrogate for oxygen-carrying capacity, almost as many patients (75) were classified anemic as were found to be anemic according to hemoglobin levels (79). Patients classified anemic based on RBC only show a significantly worse survival compared to non-anemic patients (p<0.001; Fig 2). The vast majority of clinical studies on the impact of anemia in HNSCC are based on hemoglobin concentrations [28, 29, 35–37]. Therefore, an important finding of our study is that anemia as an independent prognostic factor in surgical oncology of the head and neck should be defined as low hemoglobin concentration as well as low RBC.

### Summary

Anemic conditions of HNSCC patients are a common paraneoplastic syndrome of various causes. Unlike all other factors analyzed for their impact on survival in this study, pre- and perioperative anemia is a potential aim of therapeutic intervention. Even though in our study, perioperative blood transfusion was needed more frequently by older patients in worse physical condition suffering from more advanced stage HNSCC, subgroup analyses demonstrate the negative impact of PBT in younger patients with early stage HNSCC, too. Therefore, we conclude that a restrictive transfusion policy is appropriate in surgical oncology of HNSCC. Despite the devastating impact of anemia in HNSCC, we suggest that anesthesiologists and surgeons should restrain from correcting anemic conditions by PBT unless urgently needed.

## Supporting information

S1 File Minimal, fully anonymized set of clinical data. (XLSX)

## References

[1] Upile T, Jerjes W, Sandison A, Singh S, Rhys-Evans P, Sudhoff H et al The direct effects of stored blood products may worsen prognosis of cancer patients; shall we transfuse or not? An explanation of the adverse oncological consequences of blood product transfusion with a testable hypothesis driven experimental research protocol. Med Hypotheses. 2008 10;71(4):489–92. 10.1016/j.mehy.2008.04.027 18590945

[2] Bal SH, Heper Y, Kumaş LT, Guvenc F, Budak F, Göral G et al Effect of storage period of red blood cell suspensions on helper T-cell subpopulations. Blood Transfus. 2017 3 15:1–11. 10.2450/2016.0058-16 28488961 PMC5919838

[3] Sut C, Tariket S, Chou ML, Garraud O, Laradi S, Hamzeh-Cognasse H et al Duration of red blood cell storage and inflammatory marker generation. Blood Transfus. 2017 3;15(2):145–152. 10.2450/2017.0343-16 28263172 PMC5336336

[4] Cata JP, Wang H, Gottumukkala V, Reuben J, Sessler DI. Inflammatory response, immunosuppression, and cancer recurrence after perioperative blood transfusions. Br J Anaesth. 2013 5;110(5):690–701. 10.1093/bja/aet068 23599512 PMC3630286

[5] Shanwell A, Kristiansson M, Remberger M, Ringdén O. Generation of cytokines in red cell concentrates during storage is prevented by prestorage white cell reduction. Transfusion. 1997 7;37(7):678–84. 9225929 10.1046/j.1537-2995.1997.37797369441.x

[6] Silliman CC, Clay KL, Thurman GW, Johnson CA, Ambruso DR. Partial characterization of lipids that develop during the routine storage of blood and prime the neutrophil NADPH oxidase. J Lab Clin Med. 1994 11;124(5):684–94. 7964126 PMC4451958

[7] Jacobi KE, Wanke C, Jacobi A, Weisbach V, Hemmerling TM. Determination of eicosanoid and cytokine production in salvaged blood, stored red blood cell concentrates, and whole blood. J Clin Anesth. 2000 3;12(2):94–9. 10818321 10.1016/s0952-8180(00)00122-7

[8] Goubran HA, Elemary M, Radosevich M, Seghatchian J, El-Ekiaby M, Burnouf T. Impact of Transfusion on Cancer Growth and Outcome. Cancer Growth Metastasis. 2016 3 13;9:1–8. 10.4137/CGM.S32797 27006592 PMC4790595

[9] Wang Q, Du T, Lu C. Perioperative blood transfusion and the clinical outcomes of patients undergoing cholangiocarcinoma surgery: a systematic review and meta-analysis. Eur J Gastroenterol Hepatol. 2016 11;28(11):1233–40. 10.1097/MEG.0000000000000706 27560845

[10] Bennett S, Baker LK, Martel G, Shorr R, Pawlik TM, Tinmouth A et al The impact of perioperative red blood cell transfusions in patients undergoing liver resection: a systematic review. HPB (Oxford). 2017 4;19(4):321–330. 28161216 10.1016/j.hpb.2016.12.008

[11] Cata JP, Lasala J, Pratt G, Feng L, Shah JB. Association between perioperative blood transfusions and clinical outcomes in patients undergoing bladder cancer surgery: a systematic review and meta-analysis study. J Blood Transfus. 2016;2016:9876394 10.1155/2016/9876394 26942040 PMC4752988

[12] Mavros MN, Xu L, Maqsood H, Gani F, Ejaz A, Spolverato G et al Perioperative blood transfusion and the prognosis of pancreatic cancer surgery: systematic review and meta-analysis. Ann Surg Oncol. 2015 12;22(13):4382–91. 10.1245/s10434-015-4823-6 26293837

[13] Sun C, Wang Y, Yao HS, Hu ZQ. Allogeneic blood transfusion and the prognosis of gastric cancer patients: systematic review and meta-analysis. Int J Surg. 2015 1;13:102–10. 10.1016/j.ijsu.2014.11.044 25486261

[14] Luan H, Ye F, Wu L, Zhou Y, Jiang J. Perioperative blood transfusion adversely affects prognosis after resection of lung cancer: a systematic review and a meta-analysis. BMC Surg. 2014 5 23;14–34. 10.1186/1471-2482-14-14 24884867 PMC4057617

[15] Dicato M, Plawny L, Diederich M. Anemia and cancer. Ann Oncol. 2010 10;21 Suppl 7. 10.1093/annonc/mdq284 20943610

[16] Leifert JA. Anemia and cigarette smoking. Int J Lab Hematol. 2008 6;30(3):177–84. 10.1111/j.1751-553X.2008.01067.x 18479294

[17] Jager-Wittenaar H, Dijkstra PU, Dijkstra G, Bijzet J, Langendijk JA, van der Laan BFAM et al High prevalence of cachexia in newly diagnosed head and neck cancer patients: An exploratory study. Nutrition. 2017 3;35:114–118. 10.1016/j.nut.2016.11.008 28241978

[18] Baumeister P, Rauch J, Jacobi C, Kisser U, Betz C, Becker S et al Impact of comorbidity and anemia in patients with oropharyngeal cancer primarily treated with surgery in the human papillomavirus era. Head Neck. 2017 1;39(1):7–16. 10.1002/hed.24528 27385398

[19] Lechner M, Fenton TR. The Genomics, Epigenomics, and Transcriptomics of HPV-Associated Oropharyngeal Cancer—Understanding the Basis of a Rapidly Evolving Disease. Adv Genet. 2016;93:1–56. 10.1016/bs.adgen.2015.12.001 26915269

[20] Lydiatt WM, Patel SG, O'Sullivan B, Brandwein MS, Ridge JA, Migliacci JC et al Head and Neck cancers-major changes in the American Joint Committee on cancer eighth edition cancer staging manual. CA Cancer J Clin. 2017 3;67(2):122–137. 10.3322/caac.21389 28128848

[21] Nebe T, Bentzien F, Bruegel M, Fiedler GM, Gutesohn K, Heimpel H et al Multicentric determination of reference ranges for automated blood counts. Journal of Laboratory Medicine. 2011 4; 35(1): 3–28.

[22] Franchini M, Marano G, Mengoli C, Pupella S, Vaglio S, Muñoz M et al Red blood cell transfusion policy: a critical literature review. Blood Transfus. 2017 7;15(4):307–317 10.2450/2017.0059-17 28661855 PMC5490725

[23] von Elm E, Altman DG, Egger M, Pocock SJ, Gøtzsche PC, Vandenbroucke JP; STROBE Initiative. The Strengthening the Reporting of Observational Studies in Epidemiology (STROBE) statement: guidelines for reporting observational studies. PLoS Med. 2007 10 16;4(10):e296 10.1371/journal.pmed.0040296 17941714 PMC2020495

[24] Spivak JL. The anaemia of cancer: death by a thousand cuts. Nat Rev Cancer. 2005 7;5(7):543–55. 10.1038/nrc1648 15965494

[25] Weinstein DA, Roy CN, Fleming MD, Loda MF, Wolfsdorf JI, Andrews NC. Inappropriate expression of hepcidin is associated with iron refractory anemia: implications for the anemia of chronic disease. Blood. 2002 11 15;100(10):3776–81. 10.1182/blood-2002-04-1260 12393428

[26] Alshadwi A, Nadershah M, Carlson ER, Young LS, Burke PA, Daley BJ. Nutritional considerations for head and neck cancer patients: a review of the literature. J Oral Maxillofac Surg. 2013 11;71(11):1853–60. 10.1016/j.joms.2013.04.028 23845698

[27] Caro JJ, Salas M, Ward A, Goss G. Anemia as an independent prognostic factor for survival in patients with cancer: a systemic, quantitative review. Cancer. 2001 6 15;91(12):2214–21. 11413508

[28] Danan D, Smolkin ME, Varhegyi NE, Bakos SR, Jameson MJ, Shonka DC Jr. Impact of blood transfusions on patients with head and neck cancer undergoing free tissue transfer. Laryngoscope. 2015 1;125(1):86–91 10.1002/lary.24847 25124183

[29] Chau JK, Harris JR, Seikaly HR. Transfusion as a predictor of recurrence and survival in head and neck cancer surgery patients. J Otolaryngol Head Neck Surg. 2010 10;39(5):516–22. 20828514

[30] Opelz G, Terasaki PI. Improvement of kidney-graft survival with increased numbers of blood transfusions. N Engl J Med. 1978 10 12;299(15):799–803. 10.1056/NEJM197810122991503 357971

[31] Taniguchi Y, Okura M. Prognostic significance of perioperative blood transfusion in oral cavity squamous cell carcinoma. Head Neck. 2003 11;25(11):931–6. 10.1002/hed.10313 14603453

[32] Szakmany T, Dodd M, Dempsey GA, Lowe D, Brown JS, Vaughan ED et al The influence of allogenic blood transfusion in patients having free-flap primary surgery for oral and oropharyngeal squamous cell carcinoma. Br J Cancer. 2006 3 13;94(5):647–53. 10.1038/sj.bjc.6603013 16523195 PMC2361205

[33] Fenner M, Vairaktaris E, Nkenke E, Weisbach V, Neukam FW, Radespiel-Tröger M. Prognostic impact of blood transfusion in patients undergoing primary surgery and free-flap reconstruction for oral squamous cell carcinoma. Cancer. 2009 4 1;115(7):1481–8. 10.1002/cncr.24132 19224554

[34] Carson JL, Stanworth SJ, Roubinian N, Fergusson DA, Triulzi D, Doree C et al Transfusion thresholds and other strategies for guiding allogeneic red blood cell transfusion. Cochrane Database Syst Rev. 2016 10 12;10:CD002042 10.1002/14651858.CD002042.pub4 PMC6457993 27731885

[35] Lutterbach J, Guttenberger R. Anemia is associated with decreased local control of surgically treated squamous cell carcinomas of the glottic larynx. Int J Radiat Oncol Biol Phys. 2000 12 1;48(5):1345–50. 11121632 10.1016/s0360-3016(00)01382-1

[36] Reichel O, Panzer M, Wimmer C, Dühmke E, Kastenbauer E, Suckfüll M. Prognostic implications of hemoglobin levels before and after surgery as well as before and after radiochemotherapy for head and neck tumors. Eur Arch Otorhinolaryngol. 2003 5;260(5):248–53. 10.1007/s00405-002-0513-7 12750913

[37] Van de Pol SM, Doornaert PA, de Bree R, Leemans CR, Slotman BJ, Langendijk JA. The significance of anemia in squamous cell head and neck cancer treated with surgery and postoperative radiotherapy. Oral Oncol. 2006 2;42(2):131–8. 10.1016/j.oraloncology.2005.06.021 16146705

